# Single-nucleus atlas of circadian transcription in mouse liver and skeletal muscle

**DOI:** 10.1093/lifemeta/loag032

**Published:** 2026-10-13

**Authors:** Sijie Yang, Xiaoqing Jiang, Jing Xiang, Rongfeng Huang, Xingyong Ma, Zhihui Zhang, Jacob G Smith, Dingbang Ma, Min-Dian Li

**Affiliations:** Interdisciplinary Research Center on Biology and Chemistry, Shanghai Institute of Organic Chemistry, Chinese Academy of Sciences, Shanghai 201210, China; University of Chinese Academy of Sciences, Beijing 101408, China; Department of Cardiovascular Medicine, CEC Key Laboratory of Chronobiology and Cardiometabolic Disease, MOE Key Laboratory of Geriatric Cardiovascular and Cerebrovascular Disease, Southwest Hospital, Army Medical University, Chongqing 400038, China; Department of Cardiovascular Medicine, CEC Key Laboratory of Chronobiology and Cardiometabolic Disease, MOE Key Laboratory of Geriatric Cardiovascular and Cerebrovascular Disease, Southwest Hospital, Army Medical University, Chongqing 400038, China; Department of Cardiovascular Medicine, CEC Key Laboratory of Chronobiology and Cardiometabolic Disease, MOE Key Laboratory of Geriatric Cardiovascular and Cerebrovascular Disease, Southwest Hospital, Army Medical University, Chongqing 400038, China; Beijing SeekGene BioSciences Co., Ltd, Beijing 102206, China; Department of Cardiovascular Medicine, CEC Key Laboratory of Chronobiology and Cardiometabolic Disease, MOE Key Laboratory of Geriatric Cardiovascular and Cerebrovascular Disease, Southwest Hospital, Army Medical University, Chongqing 400038, China; Institute of Biomedicine of the University of Barcelona (IBUB), Barcelona 08028, Spain; Department of Cell Biology, Physiology and Immunology, Faculty of Biology, University of Barcelona, Barcelona 08028, Spain; Interdisciplinary Research Center on Biology and Chemistry, Shanghai Institute of Organic Chemistry, Chinese Academy of Sciences, Shanghai 201210, China; Department of Cardiovascular Medicine, CEC Key Laboratory of Chronobiology and Cardiometabolic Disease, MOE Key Laboratory of Geriatric Cardiovascular and Cerebrovascular Disease, Southwest Hospital, Army Medical University, Chongqing 400038, China

Dear Editor,

Biological clocks coordinate the spatiotemporal organization of metabolism to diurnal time cues [1]. Bulk-tissue RNA sequencing (RNA-seq) studies in mammals reveal widespread circadian (about 24 h) gene expression in mice [2–4] and nonhuman primates [5]; however, whether and how cell-type-specific organization of circadian gene expression is achieved in metabolic tissues remains unclear. Skeletal muscle and liver are crucial metabolic tissues that coordinate systemic metabolism [6–9]. Emerging evidence indicates that circadian clocks in different cell types, including myonuclei and muscle stem cells within the skeletal muscle, regulate tissue metabolism and homeostasis in a cell-autonomous manner [10–12]. In the liver, hepatocyte clocks regulate gene expression in both hepatocytes and nonparenchymal cells, including Kupffer cells [13, 14]. Here, we employed time-resolved single-nucleus RNA sequencing (snRNA-seq) to profile circadian transcription across cell types in the skeletal muscle and liver from female mice. Females were used for their relative robustness in maintaining circadian rhythms during stress versus males [15, 16].

Adult (6 months old) female mice were fed a high-fat diet (HFD) under thermoneutrality (30°C) for 8 weeks (body weight expressed in mean ± SD is 34.2 ± 4.6 g) to mimic human conditions (Western diet and thermal neutrality). After 3 days under constant darkness to unmask endogenous daily rhythms, gastrocnemius muscles and livers were dissected and snap-frozen every 3 h over a 24-h period before before being subjected to nuclei extraction and snRNA-seq, generating a data volume of 150 GB (Fig. 1a). Circadian time 0 h (CT0) is defined as the subjective time of lights-on based on preceding light-dark cycles, and tissues from two mice were pooled into a single-nucleus preparation and sequencing library at each circadian time point, resulting in eight libraries per tissue across the circadian cycle. Following an optimized workflow developed for metabolic tissues [17], a total of 72 144 and 89 973 nuclei were retained from the liver and muscle, respectively, after qua­lity control and ambient RNA removal, with comparable numbers of nuclei across all time points (Supplementary Fig. S1a). Unsupervised clustering using the Seurat pipeline identified 26 and 27 transcriptionally distinct clusters in the liver and skeletal muscle, respectively (Supplementary Fig. S1b). Nuclei from hepato­cytes accounted for the largest fraction of detected nuclei from the liver, and so did myonuclei in the muscle (Supplementary Fig. S1c). Detected genes (> 200 features per nucleus) and transcripts (> 500 unique molecular identifiers per nucleus) were comparable in size across clusters, while mitochondrial RNA content remained below 1%, indicating high-quality nuclei for downstream analyses (Supplementary Fig. S2a). Clustering was primarily driven by cell identity, as indicated by balanced representation of nuclei across samples within each cluster (Supplementary Fig. S2b).

**Figure 1:**
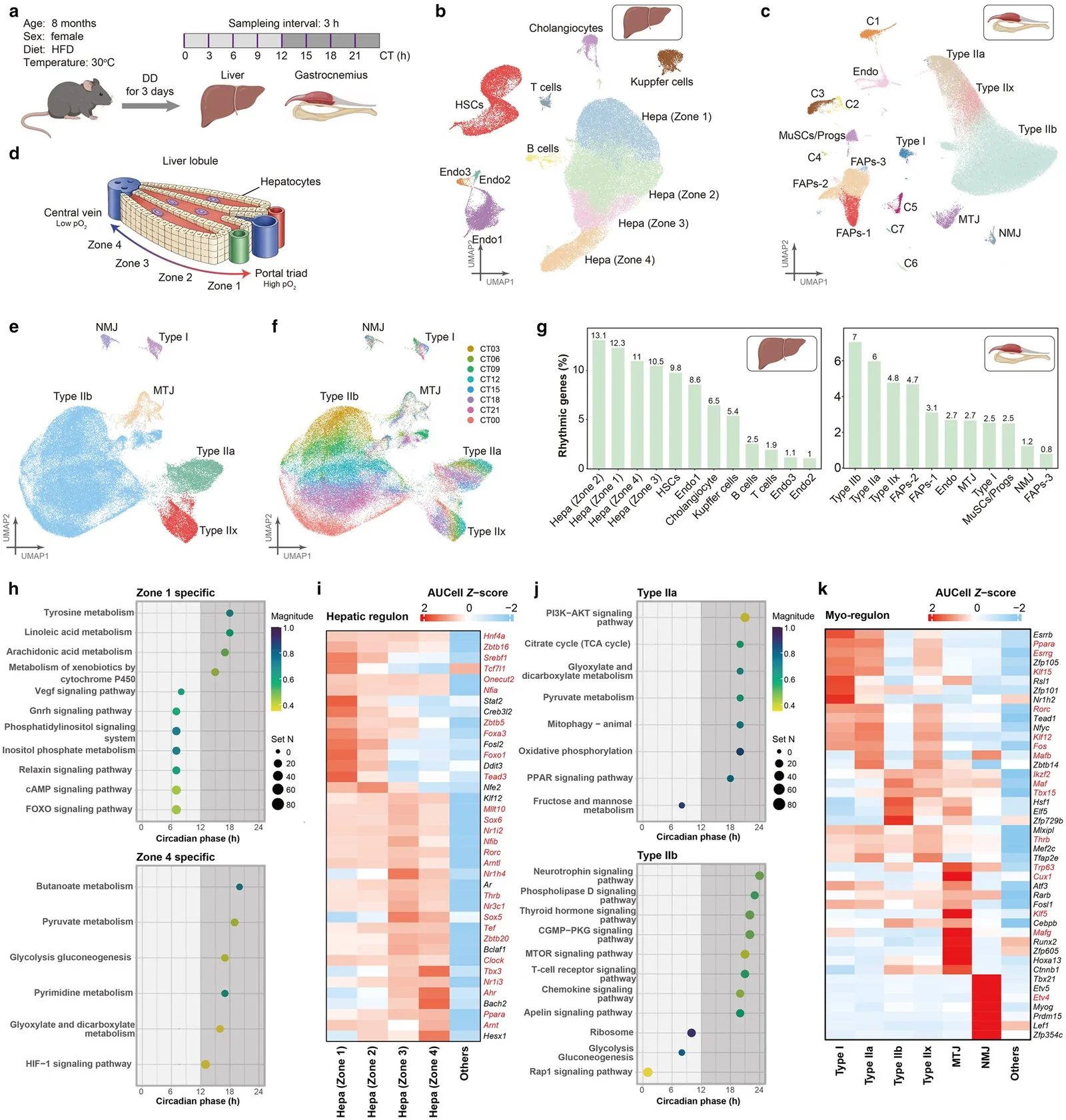
Cell-type-resolved circadian landscape of the liver and skeletal muscle in female mice. (a) Experimental design. Mice were fed 60 kcal% HFD under thermoneutrality (30°C) for 8 weeks. After 3 days of constant darkness, gastrocnemius muscle and liver tissues were dissected under dim red light every 3 h across one circadian cycle for snRNA-seq (*n* = 1 biological replicate per time point for eight time points; each biological replicate was pooled from two mice). (b) UMAP visualization of all nuclei from the liver, including zonated hepatocytes (Hepa Zones 1–4), HSCs, liver sinusoidal endothelial cells (LSECs, Endo1), venous endothelial cells (Endo2), and arterial endothelial cells (Endo3). (c) UMAP visualization of all nuclei from the skeletal muscle, including myonuclei (type I, IIa, IIb, IIx, etc.), MuSCs/Progs, FAPs, and endothelial cells (Endo). FAPs are further resolved into three subtypes: FAPs_1 (stem-like FAPs), FAPs_2 (adipogenic Ly6a^+^ FAPs), and FAPs_3 (adipogenic Ly6a^−^ FAPs). MTJ, myotendinous junction myonuclei; NMJ, neuromuscular junction myonuclei; C1, pericytes/smooth muscle cells; C2, dendritic cells; C3, macrophages; C4, Schwann cells; C5, tenocytes; C6, adipocytes; C7, neurons/glia. (d) Schematic illustration of liver zonation as a continuum along the portal (Zone 1)–central vein (Zone 4) axis of the liver lobule. (e) UMAP projection of annotated myonuclei. (f) UMAP visualization of myonuclei colored by circadian sampling time. (g) Bar plots showing the percentage of rhythmically expressed genes identified in each major cell type in the liver (left panel) and the skeletal muscle (right panel). For rhythmicity detection, each biological replicate was randomly split into two equally sized pseudo-replicates. Genes that passed RAIN (BH-adjusted *P* < 0.05) and MetaCycle (meta2d_pvalue < 0.05 and meta2d_rAMP > 0.05) from pseudo-replicates were treated as rhythmic genes. (h) Phase distribution over the 24-h cycle of KEGG pathways in metabolically distinct zonated hepatocytes (periportal Zone 1 versus pericentral Zone 4). Phases of KEGG pathways were calculated and statistically tested using the PSEA tool (Kuiper test *q* < 0.1). Magnitude denotes rhythmic amplitude; and Set N denotes the number of rhythmic genes per pathway. (i) Transcription factor analysis in metabolically distinct zonated hepatocytes, as indicated by AUCell scores (area under the recovery curve for target genes of a transcription factor or regulon in cells) using the SCENIC tool (FDR *q* < 0.05). Representative regulons were selected based on fold changes (FC > 1.1) of AUCell compared to other cell types (red denotes circadian transcription of a regulon). (j) Phase distribution over the 24-h cycle of KEGG pathways in myonuclei (glycolytic type IIb versus oxidative type IIa). (k) Transcription factor analysis in myonuclei as indicated by AUCell scores using the SCENIC tool (FDR *q* < 0.05; FC > 1.5).

Based on established marker gene sets [14, 18–20], we annotated 12 and 18 cell types in the liver and skeletal muscle, respectively (Fig. 1b and c). Hepatocytes were further resolved into four subtypes (Zones 1–4, marked by *Cryl1*, *Igfbp2*, *Cyp7a1*, and *Glul* [14], respectively), capturing a continuous transcriptional spectrum along the portal vein-to-central vein axis (Fig. 1d; Supplementary Fig. S3). Cell types in the skeletal muscle included myonuclei, muscle stem cells/progenitor cells (MuSCs/Progs), fibro-adipogenic progenitors (FAPs), and other cell types. Clustering of myonuclei revealed six distinct subtypes, including oxidative myonuclei (types I and IIa, marked by *Myh7* and *Myh2*, respectively) and glycolytic myonuclei (type IIb, marked by *Myh4*) (Fig. 1e; Supplementary Fig. S3), covering all myonuclei detectable by snRNA-seq [21, 22]. As a control identity marker, *Ryr1* was broadly enriched across all myonuclei (Supplementary Fig. S3).

Time-of-day-dependent patterns of myonuclei were clearly shown in the Uniform Manifold Approximation and Projection (UMAP) plot (Fig. 1f). Notably, type IIb myonuclei were organized sequentially based on sampling time, suggesting that time-of-day is essential for gene regulation in glycolytic myonuclei (Fig. 1f). To reveal cell-type-specific circadian gene expression patterns, nuclei at each time point were randomly divided into two equal pseudoreplicates and normalized to minimize the impact of differences in nuclear abundance. Most cell types exhibited relatively low variation across time points (coefficient of variation < 0.3; Supplementary Fig. S4). Circadian rhythmicity was calculated and statistically tested using the default parameters of two complementary algorithms, i.e., Rhythmicity Analysis Incorporating Non-parametric Methods (RAIN; average Benjamini–Hochberg method-adjusted *P-*value < 0.05) and MetaCycle (meta2d_pvalue < 0.05 and meta2d_rAMP > 0.05) [23, 24], and the phases were estimated by harmonic regression using the cosinor R package (period set to 24 h). The final rhythmic gene list was defined as the intersection of genes meeting the predefined criteria in both RAIN and MetaCycle (Supplementary Table S1). Overall, each subtype contained 1300–3000 (6%–13%) and 600–1600 (4%–8%) rhythmic genes in the liver and skeletal muscle, respectively (Fig. 1g; Supplementary Table S1). Zonated hepatocytes exhibited substantially greater circadian rhythmicity than nonparenchymal cells (Fig. 1g). Among myonuclei, those in type IIb fibers exhibited the largest number of rhythmic genes, followed by those in type IIa and type IIx fibers (Fig. 1g).

Core clock genes usually represent the unique set of rhythmically expressed genes shared among tissues in mammals. In line with this, core clock genes including *Arntl* and *Per2* were robustly rhythmic across cell types and subtypes in the liver and muscle, while some core clock genes including *Dbp* and *Cry1* were rhythmic in selected cell types (Supplementary Fig. S5a). Analysis of rhythmic cell-type transcriptomes in the muscle revealed a total of 676 rhythmic genes shared between myonuclei and FAPs, whereas 2666 and 1007 rhythmic genes were uniquely detected in myonuclei and FAPs, respectively (Supplementary Fig. S5b). In the liver, 1381 rhythmic genes were shared between hepatocytes and hepatic stellate cells (HSCs), with 3011 hepatocyte-specific and 984 HSC-specific rhythmic genes (Supplementary Fig. S5b). Myonuclei and hepatocytes exhibited the largest percentage of circadian transcriptomes in the skeletal muscle (58.3%) and liver (38.4%), respectively (Supplementary Fig. S5c). In hepatocytes, 1481 rhythmic genes were shared across different zones (Supplementary Fig. S5d). In contrast, only 29 rhythmic genes were shared among myonuclei subtypes, including core clock genes (*Arntl*, *Clock*, *Per2*, etc.) (Supplementary Fig. S5e). Together, the heterogeneity in the rhythmicity of common circadian genes beyond the core clock components suggests cell-type-specific characteristics of circadian input and output mechanisms in the skeletal muscle and liver tissues.

Phase coherence patterns among parenchymal cells and nonparenchymal cell types showed tissue- and cell-type-associated differences. In the liver, different zonated hepatocytes showed distinct phase coherence patterns relative to Kupffer cells and HSCs, whereas in the muscle, phase coherence of myonuclei of different subtypes was generally segregated from that of nonparenchymal cells, including FAPs and MuSCs/Progs (Fig. S6a). In line with these, phase patterns of zonated hepatocytes were bimodal, while nonparenchymal cells were concentrated in the active phase (Supplementary Fig. S6b). In contrast to those in the liver, cell types within the skeletal muscle, in general, exhibited evenly distributed rhythmic genes across the circadian cycle (Supplementary Fig. S6c).

Given these phase coherence patterns, it is likely that different components of signaling and/or metabolic pathways are active in a time-of-day manner in distinct cell types to adapt to periodic bioenergetic needs. To test this idea, we annotated Gene Ontology (GO) terms to rhythmic genes, and compared phases of Kyoto Encyclopedia of Genes and Genomes (KEGG) biochemical pathways (nominated by Phase Set Enrichment Analysis [PSEA]) among metabolically distinct subtypes of myocytes/hepatocytes (Supplementary Table S2). Comparison with KEGG pathways from bulk tissue transcriptomes revealed overlap of key signaling pathways such as phosphatidylinositol 3-kinase (PI3K)-protein kinase B (AKT) signaling, peroxisome proliferator-activated receptor (PPAR) signaling, mechanistic target of rapamycin (mTOR) sig­naling, and AMP-activated protein kinase (AMPK) signaling in the skeletal muscle and liver tissues (Supplementary Fig. S7); however, these pathways were active in different subtypes (Fig. 1h–j; Supplementary Fig. S8).

Many rhythmic genes were shared between hepatocytes in different zones (Supplementary Fig. S5d), along with certain signaling and metabolic pathways (e.g., AMPK/mTOR/PPAR signaling and oxidative phosphorylation) that remained robustly rhythmic at similar phases in hepatocytes of different zones (Supplementary Fig. S8). However, the identity of a subset of rhythmic output genes exhibited differences between hepatic zones (Supplementary Fig. S5d), supporting the importance of liver zonation as a factor in circadian transcriptional output [25]. At the pathway level, in Zone 1 (periportal) hepatocytes, cyclic AMP (cAMP) signaling, forkhead box O (FOXO) signaling, xenobiotic metabolism, and nutrient metabolism (e.g., amino acids and fatty acids) were active at inactive and active phases, respectively (Fig. 1h). In Zone 4 (pericentral) hepatocytes, hypoxia-inducible factor 1 signaling pathway, glycolysis, and pyrimidine metabolism were upregulated during the active phase (Fig. 1h). Regulon analysis suggested that *Srebf1*, *Foxo1*, and *Tead3* were candidate circadian regulators of Zone 1 hepatocytes, as indicated by their activity and circadian expression patterns (Fig. 1i; Supplementary Fig. S9; Supplementary Table S3). Interestingly, transcripts encoding nuclear receptors including *Nr1h4* (farnesoid X receptor) and *Ppara* were selectively enriched and rhythmically expressed in hepatocytes, compared to nonparenchymal cells (Fig. 1i), suggesting potential circadian therapeutic windows as many of these mediators are druggable [26]. We further showed that the aforementioned circadian transcription factors, including *Srebf1*, *Foxo1,* and *Nr1h4*, were regulated by the circadian clock system in the liver from a validation cohort of adult female wildtype or *Clock-*mutant mice fed an HFD for 12 weeks (Supplementary Fig. S10), providing independent bulk tissue-level evidence for clock-dependent regulation of candidate circadian regulators.

In the skeletal muscle, we found that in oxidative type IIa myonuclei, oxidative phosphorylation, citric acid cycle, and related signaling pathways including PPAR and PI3K–AKT signaling were robustly active in the late-active phase (Fig. 1j). In the glycolytic type IIb myonuclei, glycolysis/gluconeogenesis and ribosomal pathways were active in the inactive phase, which is consistent with the daily activity of biosynthesis of glycogen and proteins in the muscle (Fig. 1j). Signaling pathways, including thyroid hormone, mTOR, and apelin, were active in the late-active phase in type IIb myonuclei. Regulon analysis suggested that nuclear receptors, including *Ppara*, *Esrrg* (encoding estrogen-related receptor gamma [ERRγ]), and *Klf15*, exhibited rhythmic transcription, whose activity was linked to oxidative myonuclei such as type I/IIa (Fig. 1k). Myonuclei exhibiting glycolytic metabolism such as type IIb/IIx were associated with circadian transcription factors including *Tbx15* (Fig. 1k). At the tissue level, circadian expression of *Ppara*, *Klf15*, and *Tbx15* remained robust and was modulated by the circadian clock system in the skeletal muscle (Supplementary Fig. S10). *Esrrg* rhythm was modulated in amplitude by the circadian clock system (Supplementary Fig. S10).

In summary, our study profiles cell-type-specific circadian gene expression in key metabolic tissues in female mice and suggests the existence of spatiotemporally organized metabolism at the cellular and tissue levels (fully accessible and searchable via CircaMetDB). Our work integrates recommendations from the latest guidelines, such as thermoneutrality for metabolism research [27] and frequent sampling under constant darkness for circadian research [28], providing a resource for investigating cell-type-specific circadian transcriptional programs in metabolic tissues. Remarkably, metabolically distinct subtypes of hepato­cytes or myonuclei are associated with differential patterns of circadian metabolic gene expression and linked to circadian activities of transcription factors, including *Srebf1*, *Foxo1*, and metabolic nuclear receptors. These findings are consistent with the tissue-specific characteristics of circadian input and output mechanisms reported in bulk tissue transcriptome profiling [2, 5], as well as in fly studies on clock neurons [29–31]. Future studies should validate the cell-type-specific circadian programs in the liver and skeletal muscle, and explore how environmental cues and cell-type-specific biological clocks coordinate circadian metabolism in health and disease.

We identify several limitations of the study. First, this study used one pooled sequencing library per circadian time point and split the library into two computational pseudo-replicates for rhythmicity analysis. While it has an advantage in sampling frequency, the robustness of the rhythmic genes and pathways is limited by the number of biological replicates. Second, our data were based on female mice fed an HFD in a thermal neutral condition. Single-cell omics profiling of the liver and regenerating colon from chow diet-fed male mice under ambient temperature has been recently reported, which provides the datasets for comparative studies [32, 33]. Finally, validation studies using *Clock-*mutant mice supported circadian-clock control of selected transcription factors at the bulk tissue level; however, cell-type- and zone-specific mechanisms remain to be resolved with imaging and spatial omics in *Clock-*mutant mice.

## Supplementary Material

loag032_Supplementary_Data

## Data Availability

All data have been deposited in China National Center for Bioinformation (CNCB) under accession number CRA046535. The resource can be obtained at the website of CircaMetDB. Refer to Supplementary Table S4 for R packages.
