# Supplementary material for: Single-nucleus atlas of circadian transcription in mouse liver and skeletal muscle
Source: Life Metab. 2026 Oct 13;5(6):loag032. doi: 10.1093/lifemeta/loag032 (PMC13734757; doi:10.1093/lifemeta/loag032)
Supplement: Supplementary file 1 [file loag032_supplementary_data.zip › Supplementary_Information - tu.docx]

**Supplementary Material for**

**Single-nucleus atlas of circadian transcription in mouse liver and skeletal muscle**

Sijie Yang^1,2,‡^, Xiaoqing Jiang^3,‡^, Jing Xiang^3^, Rongfeng Huang^3^, Xingyong Ma^4^, Zhihui Zhang^3^, Jacob G. Smith^5,6^, Dingbang Ma^1,*^, Min-Dian Li^3,*^

^1^Interdisciplinary Research Center on Biology and Chemistry, Shanghai Institute of Organic Chemistry, Chinese Academy of Sciences, Shanghai 201210, China

^2^University of Chinese Academy of Sciences, Beijing 101408, China

^3^Department of Cardiovascular Medicine, CEC Key Laboratory of Chronobiology and Cardiometabolic Disease, MOE Key Laboratory of Geriatric Cardiovascular and Cerebrovascular Disease, Southwest Hospital, Army Medical University, Chongqing 400038, China

^4^SeekGene BioSciences Co., Ltd., Beijing 102206, China

^5^Institute of Biomedicine of the University of Barcelona (IBUB), Barcelona 08028, Spain

^6^Department of Cell Biology, Physiology and Immunology, Faculty of Biology, University of Barcelona, Barcelona 08028, Spain

^‡^These authors contributed equally to this work.

^*^Corresponding authors. Interdisciplinary Research Center on Biology and Chemistry, Shanghai Institute of Organic Chemistry, Chinese Academy of Sciences, Shanghai 201210, China. E-mail: [dbma@sioc.ac.cn](mailto:dbma@sioc.ac.cn) (D.M.); Department of Cardiovascular Medicine, CEC Key Laboratory of Chronobiology and Cardiometabolic Disease, MOE Key Laboratory of Geriatric Cardiovascular and Cerebrovascular Disease, Southwest Hospital, Army Medical University, Chongqing 400038, China. E-mail: [mindianli@tmmu.edu.cn](mailto:mindianli@tmmu.edu.cn) (M.D.L.)

**Supplementary Figure S1 Clustering of nuclei and cell-type annotation. (a)** Filtered nucleus number from the liver (upper panel) and gastrocnemius muscle (lower panel). **(b)** UMAP visualization of all nuclei, as measured by unsupervised clustering. (c) Proportions of annotated cell types from the liver (upper panel) and gastrocnemius muscle (lower panel).

**Supplementary Figure S2** Quality control and time composition analysis. (a) Violin plots showing quality control metrics across all identified clusters in the liver (LIV, left graphs) and gastrocnemius muscle (GAS, right graphs), as measured by the number of detected genes (nFeature_RNA), the number of unique molecular identifiers (nCount_RNA), and the percentage of mitochondrial gene expression (percent.mt). (b) Bar plots showing the percentage of nuclei from each circadian time point per cluster in the LIV (left graph) and GAS (right graph).

**Supplementary Figure S3** Violin plots displaying the expression levels of representative marker genes across distinct clusters in the liver (LIV, left panel) and gastrocnemius muscle (GAS, right panel). MTJ, myotendinous junction myonuclei; NMJ, neuromuscular junction myonuclei; C1, pericytes/smooth muscle cells; C2, dendritic cells; C3, macrophages; C4, Schwann cells; C5, tenocytes; C6, adipocytes; C7, neurons/glia.

**Supplementary Figure S4** Coefficient of variation (CV) of cell numbers across cell types in the liver (LIV, left panel) and gastrocnemius muscle (GAS, right panel). MTJ, myotendinous junction myonuclei; NMJ, neuromuscular junction myonuclei; C1, pericytes/smooth muscle cells; C2, dendritic cells; C3, macrophages; C4, Schwann cells; C5, tenocytes; C6, adipocytes; C7, neurons/glia.

**Supplementary Figure S5** Interaction of rhythmic genes among subtypes. (a) **Dot plots showing the rhythmicity of core clock genes across cell types.** Green dots indicate rhythmic expression. (b) Interaction of detected rhythmic genes between myonuclei and FAPs (upper graph) or between hepatocytes and HSCs (lower panel). (c) Bar plots showing the proportion of cell-type-specific rhythmic genes in the skeletal muscle (left panel) and liver (right panel), which collectively account for 58.3% and 38.4% of circadian transcriptomes, respectively (inset). (d) Interaction of rhythmic genes among subtypes of hepatocytes, which share 1,481 rhythmic genes among all zonated hepatocytes (inset). (e) Interaction of rhythmic genes among subtypes of myonuclei, which share 29 rhythmic genes (inset). Rhythmic genes were those that passed RAIN (BH-adjusted *P* < 0.05) and MetaCycle (meta2d_pvalue < 0.05 and meta2d_rAMP > 0.05) from pseudo-replicates. MTJ, myotendinous junction myonuclei; NMJ, neuromuscular junction myonuclei; C1, pericytes/smooth muscle cells; C2, dendritic cells; C3, macrophages; C4, Schwann cells; C5, tenocytes; C6, adipocytes; C7, neurons/glia.

**Supplementary Figure S6** Phase coherence and distribution analysis of cell-type-specific rhythmic genes.

(a) Phase coherence between cell types in the liver (left panel, 12 cell types) and gastrocnemius muscle (right panel, 15 cell types), as indicated by phase distance metrics of rhythmic genes shared between cell types. Shared rhythmic genes were identified through pairwise comparisons between cell types before subjected to phase coherence analysis. Cell types such as C2 (dendritic cells), C4 (Schwann cells), and C7 (neurons/glia) were excluded because they shared fewer than 30 rhythmic genes with fewer than half of other cell types within the skeletal muscle. The difference (Δᵢ) in phase (estimated by harmonic regression from cosinor R package) was calculated by subtraction and approximated to the interval (−12, +12 h]. Phase distance metrics between two cell types were an average of pairwise comparisons of all shared genes (i.e., mean_i_[cos(2πΔᵢ/24)]) and visualized by ComplexHeatmap. A value of 1 indicates that in-sync of all shared genes in both cell types, 0 indicates zero phase coherence, and −1 indicates anti-phase. (b and c) Phase distribution of rhythmic genes per cell type in the liver (b) and gastrocnemius muscle (c). MTJ, myotendinous junction myonuclei; NMJ, neuromuscular junction myonuclei; C1, pericytes/smooth muscle cells; C2, dendritic cells; C3, macrophages; C4, Schwann cells; C5, tenocytes; C6, adipocytes; C7, neurons/glia.

**Supplementary Figure S7** Phase set enrichment analysis of bulk tissue transcriptomes.

Time-resolved bulk tissue RNA-seq data were downloaded and reanalyzed. Phase Set Enrichment Analysis of the liver (left panel) and gastrocnemius muscle (right panel), as indicated by circadian phase, rhythmic amplitude (Magnitude), and pathway gene number (Set N), is shown. Rhythmic genes were those that passed RAIN (BH-adjusted *P* < 0.05) and MetaCycle (meta2d_pvalue < 0.05 and meta2d_rAMP > 0.05). Kuiper test *q*-value < 0.1. Source data: GSE54652 and GSE108878.

**Supplementary Figure S8** Phase Set Enrichment Analysis of zonated hepatocytes. Phase distribution over the 24-h cycle of KEGG pathways, as measured by the PSEA tool (Kuiper test *q* < 0.1) is shown.

**Supplementary Figure S9** Cell-type-specific regulon analysis by the SCENIC tool. Heatmaps show the Regulon Specificity Score (RSS) of the top enriched regulons per cell type in the liver (upper panel) and gastrocnemius muscle (lower panel), FDR *q* < 0.05.

**Supplementary Figure S10** Validation of selected transcription factors. Circadian gene expression profiling of selected transcription factors in mouse livers (upper panel) and skeletal muscle (lower panel). C57BL/6J female wildtype (WT) or *Clock* mutant (Mut) mice (21-week-old) were fed an HFD for 12 weeks (*n* = 3−4 mice per time point). After 3 days under constant darkness, samples were collected under constant darkness every 4 h for 28 h. Mutant mice had a free-running period of 28 h due to genetic mutation that resulted in skipping of Exon 19. The liver and quadriceps muscle (quadriceps is similar to gastrocnemius in physiology, metabolism and gene expression because both muscles are large hindlimb muscles with a mixed fiber-type composition) were dissected and subjected to RNA sequencing. Under this experimental scheme, differences in phase for 8−12 h (the difference in endogenous behavioral rhythms between WT and mutant mice was about 4 h, and thus lags in constant darkness for 3 days yielded a phase shift of about 12 h in theory) or loss of detectable rhythmicity between WT and mutant mice were interpreted as evidence that circadian gene transcription was modulated by the circadian clock system. Rhythmicity was calculated and tested by the CircaCompare tool. ^*^*P* < 0.05; ^**^*P* < 0.01; ^***^*P* < 0.001; ^****^*P* < 0.0001. ns, not significant. Mesor denotes the rhythm-adjusted mean level of mRNA around which a wave function oscillates; AMP, amplitude.

**Supplementary Table S1** Rhythmic genes identified in each cell type. Quantification data, summary of nuclei number, median of detected genes, and median UMIs were included.

**Supplementary Table S2** KEGG pathway analysis using PSEA tool.

**Supplementary Table S3** Transcription factor analysis using SCENIC tool.

**Supplementary Table S4** Software and R packages used in this study.
